# A Novel Approach to the Job Shop Scheduling Problem Based on the Deep Q-Network in a Cooperative Multi-Access Edge Computing Ecosystem †

**DOI:** 10.3390/s21134553

**Published:** 2021-07-02

**Authors:** Junhyung Moon, Minyeol Yang, Jongpil Jeong

**Affiliations:** Department of Smart Factory Convergence, Sungkyunkwan University, 2066 Seobu-ro, Jangan-gu, Suwon 16419, Korea; mjh7345@skku.edu (J.M.); alsduf63@naver.com (M.Y.)

**Keywords:** manufacturing process, cooperative scheduling system, job shop scheduling problem, deep Q-network, multi-access edge computing

## Abstract

In this study, based on multi-access edge computing (MEC), we provided the possibility of cooperating manufacturing processes. We tried to solve the job shop scheduling problem by applying DQN (deep Q-network), a reinforcement learning model, to this method. Here, to alleviate the overload of computing resources, an efficient DQN was used for the experiments using transfer learning data. Additionally, we conducted scheduling studies in the edge computing ecosystem of our manufacturing processes without the help of cloud centers. Cloud computing, an environment in which scheduling processing is performed, has issues sensitive to the manufacturing process in general, such as security issues and communication delay time, and research is being conducted in various fields, such as the introduction of an edge computing system that can replace them. We proposed a method of independently performing scheduling at the edge of the network through cooperative scheduling between edge devices within a multi-access edge computing structure. The proposed framework was evaluated, analyzed, and compared with existing frameworks in terms of providing solutions and services.

## 1. Introduction

Owing to the smartly changing development of the manufacturing process, the problem of efficiently handling more complex scheduling problems within the network of complex industrial sites with diversified manufacturing equipment has emerged. The job shop scheduling problem (JSP) is an NP-hard problem that requires complex job processing, which occurs in several applications [1]. Usually, to solve the scheduling problem, the manufacturing process selects and uses one of the dispatching rules (e.g., FIFO, LPT, SPT, etc.) [2]. To complement this complex process, it utilizes a local network and places a central cloud center in the factory to schedule high-volume manufacturing logistics operations. However, this may cause communication delay and latency, and if all data are centrally concentrated and a problem occurs in the cloud center and network, there is a limit that may pose a security risk. As the performance of mobile and Internet of Things (IoT) devices improves, it is possible to share work processing with the cloud center, and edge computing has attracted attention in various fields as an alternative to solve the problems of the existing central cloud method [3].

As artificial intelligence technology is gaining attention [4], previous studies have applied deep learning, machine learning, and reinforcement learning to the scheduling of congested tasks performed in industry. In particular, reinforcement learning has positive results in efficiently finding high-quality solutions to scheduling problems [5]. With the recent development of neural networks and reinforcement learning (RL) technology, various complex problems have been successfully solved by the deep-Q network (DQN), which combines deep learning (DL) and RL [6,7] and has been successfully used in the manufacturing industry [8].

A typical smart factory manufacturing industry attempts to handle scheduling in a cloud-based system. Owing to the excellent processing power of the cloud center and greater transmission speed of the network, the cloud server is stable as it handles scheduling using the latest high-performance artificial intelligence technology to handle scheduling. However, as the size of the workplace grows and the amount of data transmitted and processed grows exponentially, cloud computing environments are inefficient at processing all the data rapidly [9]. That edge computing environment is used as an alternative to cloud computing. A study for solving the JSP in a smart factory framework was conducted [10]. In this study, a method for solving the JSP using DQN in an edge computing framework ecosystem was proposed. Scheduling in an edge-computing-based environment is said to be better than processing all data at once in a traditional cloud center [11].

However, in the architecture proposed in this study, not all scheduling was performed at the edge device. When a machine’s dispatching rule is set based on the result of the neural network output layer of the cloud center, the edge device determines the scheduling of each machine according to the proposed dispatching rule and production information. In this way, the data processing area of the cloud center is allocated to the edge device; hence, data transfer between the edge device at the edge of the network and the cloud center is required. Finally, when the amount of data increases, there exists a natural delay and network security issues. Therefore, this paper proposed an architecture that performs all scheduling tasks at the edge of the network with DQN, playing the role of network monitoring and security, as well as orchestration and computing resource management at the cloud center [12].

This paper is an extension of our previous work [12], and the ultimate goal was to solve the JSP as a cooperative system rather than a single central computer. To solve this problem, we first adopted DQN and subdivided the processing area in the structure of the algorithm. Furthermore, through transfer learning, we wanted to improve the performance and help the cooperative work of the proposed architecture.

Recently, DQN, which combines deep learning and reinforcement learning, showed strong computational ability suitable for solving complex problems compared to other reinforcement learning methods. In addition, the execution of work on the edge device has low latency and improved security, and it is located at the shortest distance from the process machine at the edge of the network to speed up processing. Therefore, although an attempt was made to solve the scheduling problem using DQN in an edge-computing-based smart factory framework, there may be limitations in terms of performance to handle all tasks in an edge device. Therefore, we proposed a method of applying MEC to the smart factory architecture to perform high-performance DQN tasks at the edge of the network. Research on the application of MEC in a smart factory environment has not been actively conducted. MEC is a networking application and edge service based on intelligent interactions at the edge of the network. MEC has the advantages of low latency, high bandwidth, and a light network [13]. In MEC, we proposed an architecture that optimizes the learning speed and resource consumption by applying transfer learning to the DQN. In conclusion, after constructing the cooperative MEC ecosystem, we tried to find a new problem-solving approach by using the JSP as a sample based on DQN.

In this paper, we made the following contributions:

- As we investigated, there was no work performed with JSPs in the smart factory framework of the MEC ecosystem. This paper provided an ecosystem architecture of a smart manufacturing plant based on a collaborative edge computing framework. In addition, we proposed a cooperative computing method applicable to various manufacturing processes and smart factory operations;
- We adopted the DQN method to solve the JSP of a smart manufacturing plant. In order to determine the dispatch rules for all edge devices in the MEC-based cooperative edge computing framework, this white paper adapted the DQN to make multiple decisions to suit the requirements of the problem in question;
- We conducted comparative studies on cloud computing, a cloud–edge hybrid, and MEC. A comparison of the service operation and use such as the cost, latency, and security was performed from the perspective of service providers and users.

The remainder of this paper is organized as follows. Section 2 briefly describes MEC, DQN, and edge computing. Section 3 describes how to use MEC in the manufacturing process, introduces the modified DQN algorithm considering the MEC environment, and introduces the transfer learning method using the algorithm. In addition, we propose an architecture to which this method is applied. In Section 4, the framework evaluation analysis and experiments on the method proposed in this paper are presented. Finally, Section 6 concludes the paper.

## 2. Related Work

### 2.1. Edge Computing

As information-related technology advances, the IoT has played a key role in several fields. Interconnected devices and sensors can collect and exchange data with each other through the latest facilities in a communication network connected by tens of millions of IoT nodes [14,15]. Various IoT applications can utilize these technologies to support segmented and accurate network services to users. These IoT devices generate vast amounts of information and implement intelligent services for both service providers and users through additional preprocessing.

In a traditional cloud environment, all data must be sent to a cloud server, and the calculation results must be processed directly in the cloud, then sent back to the sensors and devices. These processes impose a great strain on the cost of data transmission on the network, especially on resources and the bandwidth. Moreover, depending on the data size, the network performance may degrade. This is a more prominent setback in smart cities [16], power grids [17], and manufacturing processes where various environments and work schedules are crucial. If data are computed in a node with a short distance from the user, the transmission time and cost are reduced. In cloud computing services, the data transmission speed is greatly affected by network traffic in addition to distance [18]. Furthermore, transmission time and power consumption costs increase according to the traffic [19]. Therefore, scheduling and data processing allocation are important issues to consider.

Edge computing is the data storage and computing used at the end of the network around the user [20,21,22]. Because the location of the edge computing nodes is located near the user, in general, the bandwidth requirements of centralized networks are greatly relaxed and traffic is reduced [23]. Moreover, it is possible to reduce the waiting time for transmission during data computing and storage operations. Numerous studies have compared the performance, strengths, and weaknesses of edge and cloud computing [3]. With the rapid increase in the number of end devices, IoT devices, and sensors, centralized cloud computing strives to meet the quality-of-service of various applications. As 5G network technology is commercialized, the edge computing method will become a key solution for solving the problems of the industry. One of the major challenges related to 5G technology is the radio access network (RAN) [24].

In the RAN, edge computing provides fast real-time information, and the RAN provides a context-aware function. Thus, by using real-time RAN information, network providers can increase the quality-of-experience (QoE) for various users [25]. Edge computing servers exist closer to users than cloud servers. Therefore, when compared to a cloud server, they provide improved quality-of-service (QoS) and relatively low latency to service users even though the computing performance is slightly inferior [26]. The requirements of an edge computing server in a typical IoT device are classified into three categories: transmission, storage, and calculation.

- Transmission:
  The total response time can be derived from the sum of the processing and transmission times. IoT devices continuously create a large amount of data, but on the contrary, the calculation request is limited [27]. In fact, large network latency cannot be accommodated, and QoS requirements cannot be satisfied. As a more specific example, when using vehicle-to-vehicle communication and vehicle-to-infrastructure communication, a faster response to the initial request is required for safety reasons. Unlike existing cloud computing, the edge computing method can service numerous distributed computing nodes that allow users to provide real-time analysis and information-collection services [28];
- Storage:
  The IoT is the source of vast amounts of data and is the most important area in the formation of big data. Therefore, the IoT needs to upload vast amounts of data to cloud- or edge-based storage. Uploading using edge-based storage shortens the transfer time. However, because the storage space of the edge node is relatively low [29] and unlike cloud computing data centers, there is no large-scale long-term storage in the case of edge storage, and because edge nodes are used in different spaces, research to update security information is required [30]
- Calculation:
  Typical IoT devices have limited power and computational resources and cannot perform complex computational tasks directly in the field. IoT devices collect data, transmit them to a more efficient computing node, and further analyze and process the data. The computing power of the edge node is superior to that of the IoT device; however, because the computing power of the edge node is limited, the scalability of edge node computing power is an issue.

An important challenge for edge computing is determining a good tradeoff between transmission latency and computing latency. A good job offloading system is required to determine whether data preprocessing is performed locally or offloaded to a cloud server. Recently, a mathematical method for achieving efficient resource allocation has been designed to determine the optimal tradeoffs [31]. Through this scheme, it is feasible to perform computational tasks locally on the final device or offload them to an edge server for execution in cloud computing. The offloading scheduling method considers a number of factors (such as the execution state of the local processing unit and the task buffer queuing state). This approach can reduce the average power consumption of the final device and the average latency of individual tasks.

### 2.2. Multi-Access Edge Computing

**Figure 1:**
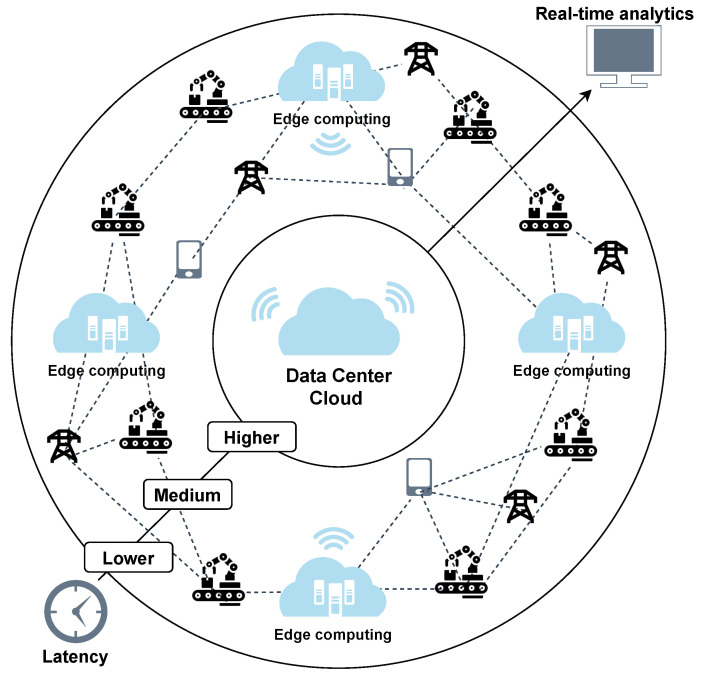
MEC framework.

In edge computing, when data are transmitted to a cloud computer, the transmission speed based on the traffic of the edge computing is unstable compared to cloud computing. In addition, it is difficult to solve data processing and scheduling allocation in an edge device. Therefore, we proposed a method to solve the two problems mentioned above by using MEC, which has recently attracted attention. The goal of MEC is to extend cloud computing capabilities to the edge of the network, which minimizes unstable network congestion and optimizes resources, improves the overall performance of the network, and provides enhanced user experience. Figure 1 briefly describes the structure of MEC.

Edge computing is generally adjacent to the user as in this figure, so it has low latency and allows for persistent connections. Furthermore, using the edge of the network increases computing power. Moreover, during high traffic on the edge network, data can be offloaded to the cloud to maintain a fast and reliable connection, as shown in Figure 1.

MEC is a major technology recognized as the driving force behind 5G networks. Compatible with the current 4G network environment, MEC plays a very prominent role as a helper in 5G systems developed with the spread of IoT. MEC technology provides cloud computing services within the RAN and provides a mobile information technology service infrastructure at the edge of the network adjacent to mobile users, which provides high bandwidth, low latency, and wireless capabilities that can be used by QoS-optimized platforms and applications. It features real-time access to the network information. The edge may refer to both the data center and the base station itself (e.g., a radio network controller (RNC) and an Evolved Node B (eNB)) adjacent to the wireless network.

When a new user enters the MEC environment, an additional connection is performed by opening the RAN edge. This allows for rapid and flexible services and deployment of applications.The MEC method benefits not only operators who can run applications close to mobile users at the edge of mobile networks, but also other operators.

The architectures of the ETSI, MEC, ISG, and MEC frameworks were released in December 2014, and work was performed to improve the performance to be able to host third-party applications in a multivendor environment [32]. It is a great advantage of edge computing to send and receive data in real time with reduced latency in proximity to the user’s device. MEC is a networking edge service and application that is based on intelligent action at the equipment terminal. As device-to-device (D2D) communication technology has expanded to various industrial fields, convenient resource allocation is possible by utilizing the base station of a smart factory [33].

Convergence between various types of data is required for intelligent control and data collection in complex industrial processes. MEC can reverse the data-fusion function at the edge of the network. In peripheral network devices, edge computing makes the best use of the device’s sensor resources, and the information obtained from them is combined according to algorithms and defined optimization criteria. This process can utilize knowledge-based self-learning mechanisms to generate responses, perform dynamic inference, and improve the consistency of the analysis and interpretation of detected situations. MEC nodes can apply techniques such as frequency analysis, wavelet analysis, and time series analysis to extract functions from sensor signals. Machine learning models or artificial intelligence can be used in edge nodes to make predictions using updated knowledge bases and feature data. Thus, intelligent decisions can be made at the edges of the network. The collection and implementation of finely separated data can be easily performed on peripheral IoT devices, and multiple streams of original data can be directly combined.

In addition, the manufacturing process can be visualized thoroughly using advanced data visualization technology. This technology allows the system to predict errors and perform more rapid active maintenance. Because the system is aimed at handling short-term or real-time data analysis, MEC can support the business’s processing of real-time information more quickly. This approach can improve data security and privacy and reduce the burden on the network. However, cloud computing does not focus on short- or real-time data processing from the outset. Table 3 shows the comparison index among the characteristics of the cloud, cloud–edge hybrid, and MEC-based edge computing methods. In ETSI, MEC changed its name to ess edge computing instead of mobile edge computing to apply it to various areas beyond WiFi and mobile fixed-access technology [34].

### 2.3. Job Shop Scheduling Problem

**Figure 2:**
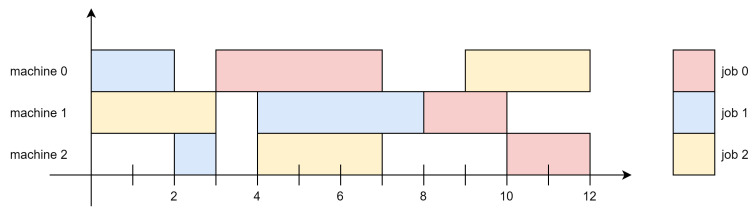
Job shop scheduling problem.

The workplace scheduling problem can be understood as shown in Figure 2. Assuming that there are n jobs in J $=\;\left\{J_{1},\;J_{2},\cdots ,J_{I},\cdots ,J_{n}\right\}$, each job consists of job $J_{i}=\;\left\{O_{i1},O_{i2},\cdots ,O_{il},\cdots ,O_{im}\right\}$, and m machines in $M=\;\left\{M_{1},M_{2},\cdots ,M_{j},\cdots ,M_{m}\right\}$ each have to process m operations. Each operation has a designated processing machine and a corresponding processing time, and there is a limit on the processing order for the same job. In the JSP problem, a machine-specified matrix $O_{M}$ and a processing time matrix $O_{T}$ are provided. The machine designation matrix is $OM=\;\left\{m_{il}|m_{il}=M_{1},M_{2},\cdots ,M_{m}\right\}$(i=1,2,…,n,l=1,2,…,m), and the processing time matrix is expressed as $OT=\;\left\{t_{il}|t_{il}>0\right\}\left(i=1,2,\ldots ,n,\;l=1,2,\ldots ,m\right)$. Operation $O_{\mathrm{il}}$ is processed by machine $m_{\mathrm{il}}$, and $t_{\mathrm{il}}$ is the processing time of $O_{\mathrm{il}}$.

If three different jobs with multiple jobs need to be processed on three machines, each job has a specified order, and the job must be processed after the previous job is completed. For example, Job 0 must first pass through Machine 0, then Machine 1, and, finally, Machine 2 to obtain the result, and the order of each operation is not exactly the same. The final goal is to efficiently allocate three jobs to be processed in the machine to minimize the maximum time for all jobs to be allocated and completed [35].

Then, the JSP is set as a conditional optimization task. The time to complete the last execution of all jobs, that is the makespan, should be allotted an efficient operation that can make it the minimum. It should be considered that each machine can process only one job at a time, and there is a restriction on the order of processing of integrated jobs. As previously mentioned, the next job cannot be executed until the previous job is completed, and when the schedule allocation of each job to each machine is determined, the completion time of each operation $O_{\mathrm{il}}$ can be known. The completion time of all tasks is the maximum time in the same system, plus the processing time of the previous task. The makespan value is calculated based on this rule.

Our proposal aimed to solve the JSP in a smart factory framework. That is, if orders from multiple customers each containing J or more jobs are given, each job has a set of jobs that must be processed in the system in a specific order, and to simplify this problem, each job must be processed once on each machine. After executing a series of J jobs in the JSP related to this study, the MEC of the factory must find a method (DQN) to determine the start time of each job. It is processed by the system so that the makespan (maximum time to complete) is minimized. Then, each edge device receives the calculated scheduling result and executes the calculation result on the controlled machine.

### 2.4. Applying AI Technology to Scheduling

Time-consuming and complex processes such as semiconductor manufacturing have various challenges in achieving ideal productivity owing to the complexity of the manufacturing process [36,37]. The metaheuristic algorithm-based (e.g., genetic algorithm) method [38], which has been frequently adopted in recent years, has several disadvantages. It is difficult to adapt it to a dynamic environment, and it requires a complex design process. Accordingly, numerous studies have attempted to combine scheduling and reinforcement learning (for example, particle swarm optimization (PSO) [39] and ant colony optimization (ACO) [40]) and simulation annealing [41]. Deep reinforcement learning (DRL), which combines the advantages of deep learning and reinforcement learning, solves the scheduling problem of cloud manufacturing using an efficient and intelligent approach.

The DQN, which has recently gained attention, has presented the use of DRL to solve complex problems and has shown improved experimental results [6,7]. AlphaGo’s success in particular attracted great attention to the DQN [42]. The DQN was mainly adopted in applications that play Atari games in previous studies [43], and in recent studies, it has been used as a way to solve the JSP [8]. The JSP is NP-hard and is a very difficult computational problem. The actual sequence of work performed in the factory is complex; therefore, in the field, factory personnel apply a number of dispatching rules to determine the work schedule for each system. Dispatching rules are used to determine the order of work because they are simple and easy to implement, easy to understand, and provide good management insights [44]. In fact, many manufacturing plants use dispatching rules to solve work schedule problems [45]. Therefore, a method for efficiently solving complex JSPs using a DQN in an edge computing environment was studied.

## 3. Scheduling System Using the MEC Structure

**Figure 3:**
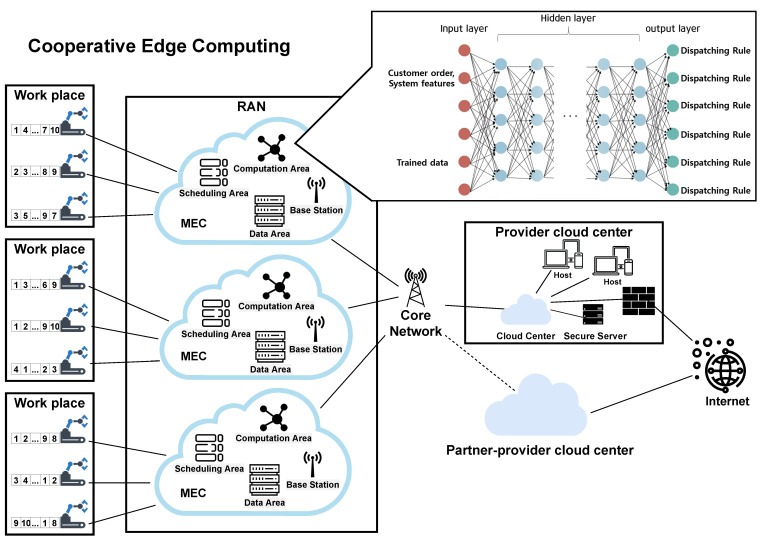
Smart factory manufacturing process service provider’s cooperative scheduling solution framework.

In this study, the DQN approach was used to solve the JSP through the MEC structure in the manufacturing process. When scheduling is performed, a framework that can be handled in collaboration with the surrounding MEC infrastructure was proposed. Figure 3 is a real-world system framework for solution services targeting manufacturing companies whose service providers are part of the infrastructure [12].

The role of the cloud center is to use the resources provided by MEC to monitor the network or perform overall system management tasks to be centrally handled, such as job orchestration, traffic offloading, and system security. The battery issue that attracts attention in the mobile environment was not applied because power is not a limitation in the manufacturing industry. Therefore, many of these problems disappear when applied to industrial sites. MEC can reduce latency by offloading complex computational tasks to nearby computing resources contained in the RAN environment. In addition, the DQN method can achieve maximum performance by focusing on computing power. The MEC is a base station (BS) that examines packets that have accessed the network. If a potential security problem is identified based on the normal and abnormal patterns classified in advance, the abnormal packet is transmitted to the backbone (cloud computer). Then, it is checked in the cloud so that subsequent processing can be performed.

In edge devices, sufficient computing resources must be secured to perform all scheduling tasks. To this end, we proposed a process for performing cooperative scheduling between edge devices in an MEC ecosystem. First, the data are collected through a working machine. It then transmits the data to the edge of the MEC via an IoT device. The components of the MEC are as follows: First, there is a BS area in charge of communication between the inside and outside. Second, there is a computation area that derives the dispatching rule through an NN that applies transfer learning. Third, there is a scheduling area that determines the scheduling of all machines based on the derived dispatching rule. Finally, it is divided into a data area for storing prelearned data used for transfer learning or various data necessary for scheduling and different applications included in the MEC environment.

The BS blocks all packets detected when accessing the MEC and sends information about the packet to the cloud center. The computation area is responsible for all the NN operations such as NN propagation and backpropagation applied to the training data. In the scheduling area, it is in charge of a task that directly affects scheduling based on the dispatching rule. A description of the scheduling method is presented in Section 4.

We proposed a method for a solution developer that provides an integrated service environment in the smart factory manufacturing process to provide optimized JSP services to customers. First, the customer’s MEC network is formed based on the service provider’s RAN network. A huge infrastructure can be built through a core network between the MEC network and the cloud center (service provider). It also prepares transfer learning based on pre-optimized training algorithms and system functions. These pretrained data are fed into each customer’s MEC data area. These data may be updated in the future or lead to better learning outcomes according to changes in the environment of the MEC.

Each MEC edge is connected to the customer’s manufacturing process workplace. Based on this, it collects data and supports self-scheduling. In this case, confidentiality is ensured because the cloud center does not take crucial and sensitive key information during the manufacturing process. The MEC assigns a schedule to all systems. This will continue to drive the upgraded function through the system, so it can be shared on the core network and used by the service provider or another MEC. It is also possible to receive excellent learning results from other customers and update them in their own system. This method is effective at mitigating the computing resource shortage of edge devices. This is because better scheduling results can be obtained faster based on the excellent training data in advance. However, the service provider providing the solution should build the MEC environment and scheduling structure of customers as similarly as possible. Through similar research activities and simple simulations, we found that the learning process for each MEC was complex, and the scheduling results were worse if they were not consistent with each other. We proposed DQN transfer learning through a detailed explanation.

In the overall framework, it does not end with a single service network; it can be extended to networks of other service providers. We shared excellent learning methods with the company by opening the core network to other cloud centers with which we signed prior agreements. This can also enhance security by exchanging information about anomalous packets and forming a wider network. Other service providers provide more opportunities and more information to small businesses that have difficulty in forming their own manufacturing process systems. This gives them excellent results found by their own company. In this case, the service provider should aim to build a service network throughout the manufacturing process.

## 4. Scheduling Methodology

### 4.1. Scheduling Method Based on DQN

We proposed a new method in which scheduling is performed using only edge computing in the existing cloud–edge hybrid method. This method uses an approach based on the DQN algorithm. The notations used in this paper are summarized as follows.

- *M*: number of machines;
- *J*: number of jobs;
- $P_{jm}$: processing time of job j at machine m;
- $F_{m}$: completion time of machine m;
- $C_{j}$: completion time of job j;
- $\mu_{M}$: utilization ratio of machine m;
- $\sum_{j}\sum_{m}P_{jm}$: sum of all processing times;
- $C_{\max}={\mathrm{Max}}_{j}C_{j}$: makespan.

The DQN, which performs all scheduling areas only in the MEC structure, is the same as Algorithm 1 and is divided into an input layer, hidden layer, and output layer.

The order of development of the proposed method is as follows. First, transfer learning is performed by importing a pretrained model from the computation area of the MEC for fast learning using fewer computing resources. Then, the customer order and system features provided in the work place are entered into the NN, and the NN propagation is performed.

Then, according to the result of the NN output layer, the dispatching rule of all machines is determined using the ϵ-greedy policy. The ϵ-greedy policy allows the probability of selecting an arbitrary action in the initial epoch when starting learning, but gradually decreases this probability as the number of epochs increases [8].

Subsequently, in the scheduling area, the scheduling of each machine is decided based on the dispatching rule determined in the computation area and the actual production flow of the system. When determining and monitoring all job schedules, waiting for a certain period of time is necessary to prevent the start time of the job processed in the machine from being earlier than the end time of the job processed in the previous task. The MEC recovers the start and end times of the jobs in which these issues occur.
**Algorithm 1** The DQN performed only in the MEC (N customer orders).1:**for** i = 1,2, …, N **do**2:    Consider *J* jobs in customer order i. Assign random or certain dispatching ruleto each machine.3:    Compute system features $\bar{F}$, $C_{max}$, $\bar{Q}$, $\left\{\mu_{m}\right\}$, and $\left\{\bar{O_{m}}\right\}$.4:    Create a state based on $\left(J,C_{max},\mu_{1},\mu_{2},\ldots ,\mu_{M}\right)$ in the Q-table.5:    The NN consisting of customer orders, system features, input neurons, and outputneurons.6:    **for** epoch number t = 1, 2, …, T **do**7:        Propagation of the NN in the computation area.8:        The computation area uses an ϵ-greedy strategy to determine the dispatching rules   of all machines and transmit the information to the scheduling area.9:        The scheduling area determines the scheduling of jobs in the controlled machines.10:        The computation area oversees the scheduling results of all machines and   then repairs the scheduling results.11:        The computation area creates a state based on $\left(J,C_{max},\mu_{1},\mu_{2},\ldots ,\mu_{M}\right)$ in the   Q-table and updates the Q-table in the output layer using   $q_{m}^{\prime }=\left[q_{m}+\left(\mu_{m}^{\prime }-\mu_{m}\right)/\mu_{m}\right]+\gamma$$\cdot \mathrm{Ma}x_{a}Q\left[s^{\prime },a\right]$.12:        The computation area updates the system features:   $\bar{F},C_{max},\bar{Q},\left\{\mu_{m}\right\},\;and\;\left\{\bar{O_{m}}\right\}$13:        The computation area updates the hidden layer by the loss $E\left[{\left(Q_{m}^{\prime }-Q_{m}\right)}^{2}\right]$.14:        The computation area decreases a probability value ϵ by ϵ-diff.15:    **end for**16:**end for**

The proposed DQN characterizes the system state of the NN through certain features and updates the Q-table to record the best output quality of all state transitions discovered up to this point. The definition of the characterization state is $\left(J,C_{\max},\mu_{1},\mu_{2},\ldots ,\mu_{M}\right)$, and job *J* is one of the input parameters of the NN and affects the performance. Therefore, it can be concluded that *J* is included in this state. $C_{\max}$ is the total time required, the makespan. In addition, because the machine feature were captured by the machine’s utilization $\mu_{1},\mu_{2},\ldots ,\mu_{M}$, *M* was not included in the state. Because there are too many values applicable to each of these features, the total number of states may be excessively large, so $C_{\max}$ is set in five classes [0, 0.5W], [0.5W, 0.7W], [0.7W, 0.9W], and [0.9W, 1.0W]. W is the sum of all processing times. This can be defined as $\sum_{j}\sum_{m}P_{jm}$. $P_{jm}$ is the job processing time of job j on machine m. After the scheduling results are obtained, $\left(J,C_{\max},\mu_{1},\mu_{2},\ldots ,\mu_{M}\right)$ can be obtained, and thus, the state can be characterized. If this state is a new state, the Q-table is extended to the new state. That is, new rows and columns are added for this state, and all new entries are initialized to zero vectors. If the original state is s and the new state is $s^{\prime }$, it can be defined as $Q\left[s,s^{\prime }\right]=\left(q_{1}^{\prime },q_{2}^{\prime },\ldots ,q_{M}^{\prime }\right)$, where each m∈{1,2,…,M}. The element $q_{M}^{\prime }$ is calculated using Equation (1).

(1) $$q_{m}^{\prime }=\left[q_{m}+\left(\mu_{m}^{\prime }-\mu_{m}\right)/\mu_{m}\right]+\gamma \cdot {\mathrm{Max}}_{a}Q\left[s^{\prime },a\right]$$

Gamma (γ) is the learning rate. When the output layer is updated, the new system features $\bar{F},C_{\max},\bar{Q},\;\left\{\mu_{m}\right\},\bar{O_{m}}$ are calculated and sent to the neurons of the NN input layer according to the scheduling results, which will be used for the next run of propagation. Finally, at the end of the epochs, the loss of the predicted result based on the Q-value in the computation area is calculated as Equation (2). $q_{m}^{\prime }$ is the Q-value calculated by applying Equation (2) to the loss calculation.

(2) $$Loss=E\left[{\left(q_{m}^{\prime }-q_{m}\right)}^{2}\right]$$

**Figure 4:**
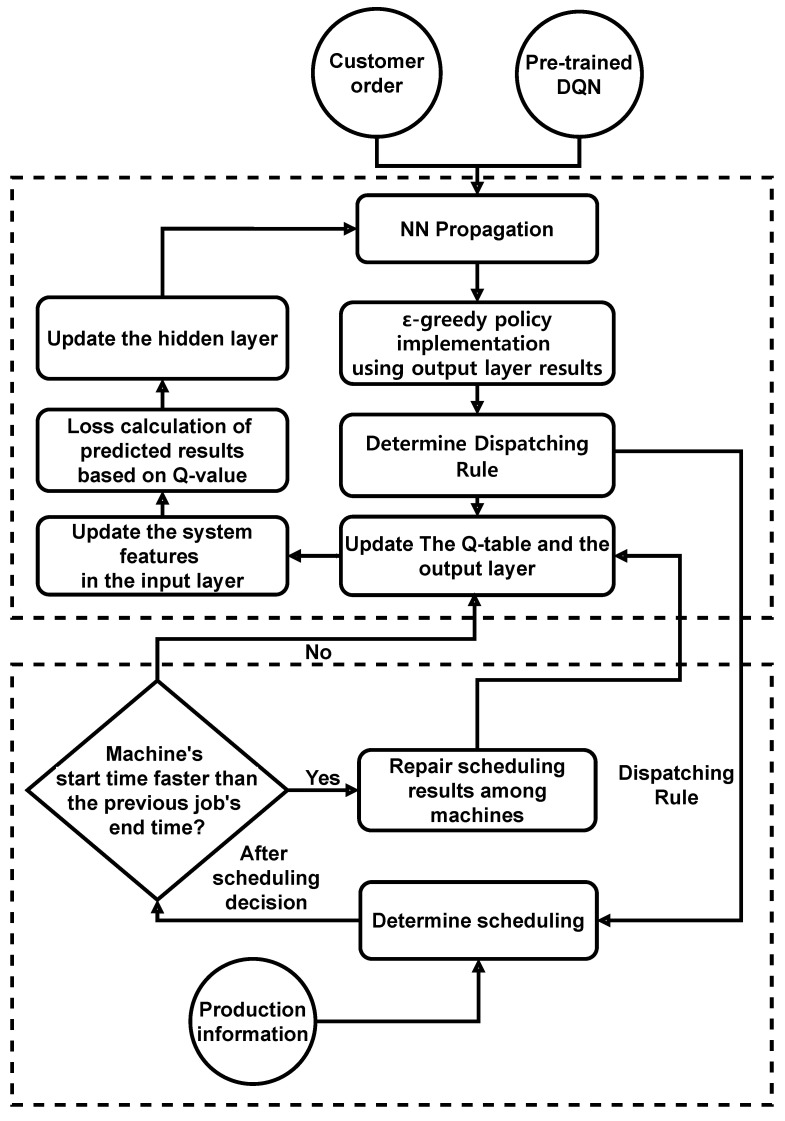
Job shop scheduling problem.

When an action is taken in the current state *s*, the next state to reach the corresponding network is $s^{\prime }$. The Q-function value of the action that can be taken in this process is set to $q_{m}^{\prime }$. $q_{m}$ is the result of the NN output layer. Then, backpropagation is performed to update the weight of the hidden layer using the corresponding loss value in the NN computing area of the MEC. Figure 4 shows the flowchart for the DQN’s execution [12].

### 4.2. Transfer Learning to the DQN

The ability to learn efficiently by transferring previous knowledge to new situations can be useful for intelligent agents. In particular, transfer learning of excellent methods is essential for cooperatively solving JSPs within MEC. For this, we adopted the “actor–mimic” method. This method generalizes knowledge to new domains, and the agent learns to perform multiple tasks. It uses deep reinforcement learning and model compression techniques. A single policy network is trained, using the guidance of multiple expert teachers, and learns the flow and method of acting in a set of individual tasks. It is possible to generalize new tasks from expressions learned in deep policy networks without prior expert guidance, thus speeding up learning in new environments as well [46]. While this is generally applicable to a wide range of problems, we applied it when using the DQN approach for solving scheduling problems.

Using a set of preprepared source data $\left(S_{1},\ldots ,S_{N}\right)$, the first goal was to obtain a single multitasking policy network that can mimic expert scheduling optimized to the maximum. To train this multitasking policy network, we used the guidance of the expert DQN network $\left(E_{1},\ldots ,E_{N}\right)$. Here, $E_{i}$ is the optimized expert state of the source task $S_{i}$. Then, the Q-value and the matching squared loss between the target MEC becoming a student and the MEC becoming an expert are defined. The range of the expert value feature can vary greatly from task to task, and we first converted the Q-value using softmax and match the policy. When softmax is used, the two outputs are limited in unit intervals, so the external influence of various scales according to each expert Q feature is reduced, and stability during learning is improved. The student MEC can use softmax to focus more on imitating the behavior chosen by the guiding expert MEC, as observed in each state where the exact value of the state is less important. It is called “actor–mimic” because it is an actor that imitates the policy that is the decision of the expert. First, each expert DQN is transformed into a policy network using the Boltzmann distribution defined for the Q-value output.

(3) $$\pi_{E_{i}}\left(a|s\right)=\frac{e^{\tau^{-1}Q_{E_{i}}\left(s,a\right)}}{\sum_{a^{\prime }\in A_{E_{i}}}e^{\tau^{-1}Q_{E_{i}}\left(s,a^{\prime }\right)}}$$

Here, τ is the temperature parameter and $A_{E_{i}}$ is the task space used by expert $E_{i},$$A_{\varepsilon_{i}}\subseteq A$. Given the state of the source task $S_{i}$, the policy target for the multitasking network is defined as the multitasking network, which is the cross-entropy between the expert network policy and the current multitasking policy.

(4) $$L_{\mathrm{policy}\;}^{i}\left(\theta \right)=\sum_{a\in A_{E_{i}}}\pi_{E_{i}}\left(a|s\right)\log\pi_{\mathrm{AMN}\;}\left(a|s;\theta \right)$$

Here, $\pi_{\mathrm{AMN}}\left(a|s;\theta \right)$ is a multitasking actor-mimic network (AMN) policy parameterized by θ. Unlike the Q-learning goal, which recursively depends on the target value, we now have a stable supervisory training signal (expert network output) that guides the multitasking network. To obtain training data, the output of an expert network or AMN task can be sampled to generate the trajectory used for the loss. During training, the AMN sampling results in the best results, and later, when the AMN is learning from an expert or the AMN, when the AMN is at least approximate to a linear function, it is verified that it converges to the expert policy using policy regression loss.

θ and $\theta_{f_{i}}$ are the parameters of the AMN and i-th feature regression network, respectively. When learning this goal, the error is completely backpropagated at the output of the feature regression network through the AMN layer. In this way, the feature regression goal puts pressure on the AMN to compute a feature that can predict the expert features. Justifying this goal is that in the case of a complete regression from multitasking to an expert feature, all the information of the expert feature is included in the multitasking feature. If a separate feature prediction network $f_{i}$ is used for each task, the multitasking network can prevent the identification problem while having a feature dimension different from that of the expert. Empirically, the main advantage of the feature regression goal is that it can improve the performance of transfer learning in certain goal tasks. Combining the two regression goals, the actor–mimic goal is defined as Equation (5).

(5) $$L_{\mathrm{ActorMimic}\;}^{i}\left(\theta ,\theta_{f_{i}}\right)=L_{\mathrm{policy}\;}^{i}\left(\theta \right)+\beta *L_{\mathrm{FeatureRegression}\;}^{i}\left(\theta ,\theta_{f_{i}}\right)$$

where β is a scaling parameter that controls the relative weights of the two targets. One can think of the policy regression goal as a teacher (expert network) who directs students (AMN) how to behave, and the feature regression goal is similar to a teacher who instructs students why they should behave.

Now that we have a network training method that is an expert in all source tasks, we can proceed to transfer the source work knowledge to a new, but relevant target task. To transfer to the new task, the final softmax layer of the AMN is first removed. Then, the AMN weight is used as an instantiation of the DQN to learn about the new target task. The pretrained DQN is then trained using the same training procedure as the standard DQN. Multitasking pretraining can be viewed as initializing the DQN as a set of functions that are effective at defining policies in related tasks. If the source and target tasks share similarities, some of these pretrained functions may be effective in the target task. We proposed an architecture for efficient scheduling tasks that perform transfer learning by targeting a predefined series of the DQN in the MEC within the same network. The DQN parameterizes the state–action value function using a deep convolutional neural network for the pixel input.

The DQN was trained in combination with certain tricks that stabilized the training of the network based on a learning model that was pretrained on other edge devices. It also maintained the same network architecture and hyperparameters for all scheduling tasks. Each network had similarities in the scheduling tasks, but was limited to learning only one task at a time. A network trained to perform multiple scheduling tasks can generalize knowledge between scheduling tasks, and the network can achieve one compact state representation by using the similarity between tasks. The network can speed up learning by showing a transition to a new target task after being trained on sufficient source tasks. Based on such transfer learning, we proposed to perform scheduling work content sharing through the DQN in a huge manufacturing solution environment.

## 5. Performance Analysis

In this section, we evaluated the performance of the proposed DQN for all jobs by considering the relevant JSP in terms of the manufacturing period. In previous studies, experiments that attempted to solve the JSP in an MEC-based manufacturing environment were not actively conducted. Our experiment was conducted in two tracks, each focusing on quantitative and qualitative evaluations. First, we analyzed the performance of the proposed method using different parameter settings. Then, a quantitative evaluation was performed to compare the performance of the dispatching rule (SPT, LPT), the traditional DQN, and the DQN using the proposed transfer learning. Finally, we compared and evaluated the proposed cooperative MEC framework with cloud and existing cloud–edge hybrid methods.

### 5.1. Experimental Environment

The experiments and training performed in this study were conducted in an environment with an Intel Xeon 2.2GHz CPU, 1, 25GB RAM, and an NVIDIA V100 GPU with a learning rate of $10^{-6}$. Furthermore, we used PyTorch Version 1.7.0 to build the model. Models were defined with the torch.nn module and torch.optim module, and GPU-accelerated features were used during training and testing using the torch.cuda module.

The experimental section was performed on the JSP benchmark datasets in [47]. These dataset contain instances of the titles “Lawrence (la)”, “Demirkol, Mehta and Uzsoy (dmu)”, “Fisher and Thompson (ft)”, and “Applegate and Cook (orb)”, and several others used in this experiment were included. Instances had different numbers of machines (M) and jobs (J). The parameters used in this study were divided into two types. (1) Parameters of the ϵ-greedy policy: In the ϵ-greedy policy, the values for the parameter ϵ were ϵ = 0.9 and ϵ-diff = 0.02, and they were reduced by ϵ-diff for each epoch of the algorithm. (2) Learning rate for Q-table update: The ratio of training data and test data for the existing DQN was set to 2:1. Furthermore, the number of neurons in the hidden layer of the NN was 64, the learning rate 0.001, and the discount rate (γ) 0.7. The parameters used in this study were as described above, and they are summarized in Table 1.

The proposed transfer-learning-based DQN learns based on pretrained data. We trained a predictive model using the training data and evaluated the model performance using the test data. The number of machines in the factory does not change often, so it was considered fixed, and the experiment was conducted. Accordingly, a fixed number of machines M and J were used for the evaluation of the experiment. In addition, to obtain high prediction efficiency, experiments and comparisons were performed using customer orders with a fixed number of tasks. The proposed model can be applied to a different number of machines and tasks than those in our experiments. It was assumed that the model to perform transfer learning and the structure of the learning model were the same in order for learning to proceed quickly and to obtain high performance.

### 5.2. Convergence and Comparative Analysis

In this section, a convergence analysis was performed for model training by using the proposed method by setting the parameter ϵ-diff factor, considering the number of machines, the number of tasks, and the ϵ-greedy policy. When the DQN algorithm was applied to the datasets used in the experimental process of this paper, the actual execution time per epoch was very short. Makespan means the total processing time, and the unit of time was ms. According to the calculation, the scheduling problem took about 20 min to perform 1000 epochs. Of course, in the industrial field, there are many things to consider, and more real-time work needs to be performed faster and faster. However, our point was to change the scheduling approach from standalone to collaborative, independent of computing power, and we wanted to apply it to our manufacturing process.

**Figure 5:**
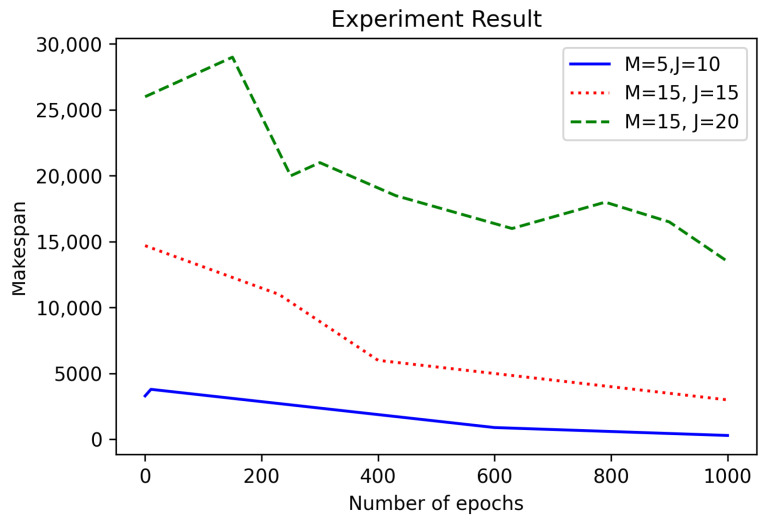
Convergence analysis according to the number of M and J.

Figure 5 shows the convergence analysis for different combinations of numbers *M* and *J*. The parameters were the “la” instances with M=5 and J=10 and the “dmu” instances with M=15 and J=15 or 20. In Figure 5, the higher the number of machines or jobs, the slower the convergence rate is. Through this experiment, we were able to confirm the makespan derived from each dataset. In addition, we confirmed that the convergence speed of the makespan slowed down when the number of machines and jobs increased, so it was possible to clarify the variables to consider when to schedule.

**Figure 6:**
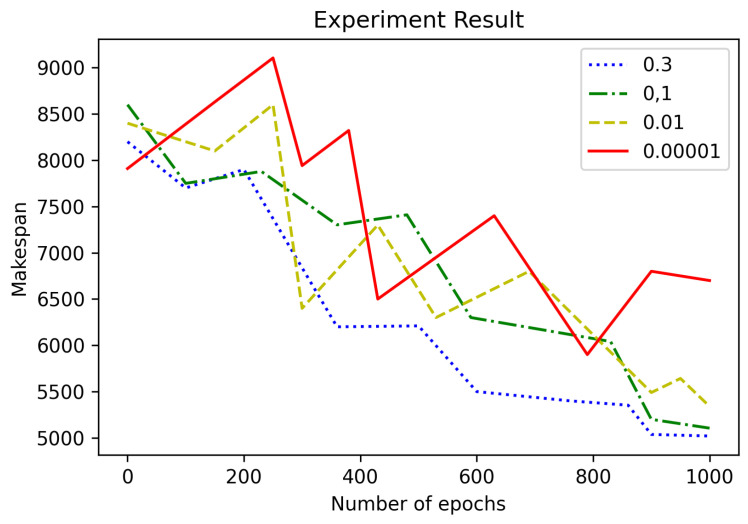
Convergence analysis according to the difference in parameter ϵ-diff.

Additionally, we compared and analyzed the performance of the DQN and the DQN that performed the transfer learning proposed in this study. Figure 6 shows a convergence analysis for parameter ϵ-diff. In Figure 6, we can notice that the method using ϵ-diff =0.00001 converged slowly. The smaller the value of ϵ-diff was, the more randomly it performed, and it converged slowly. ϵ used a value between 0.0 and 1.0, usually decreasing gradually as the search progressed. In the text, this was called ϵ-diff, and it is called epsilon decay. We used epsilon to determine how random we wanted it to be when performing the initial work.

**Figure 7:**
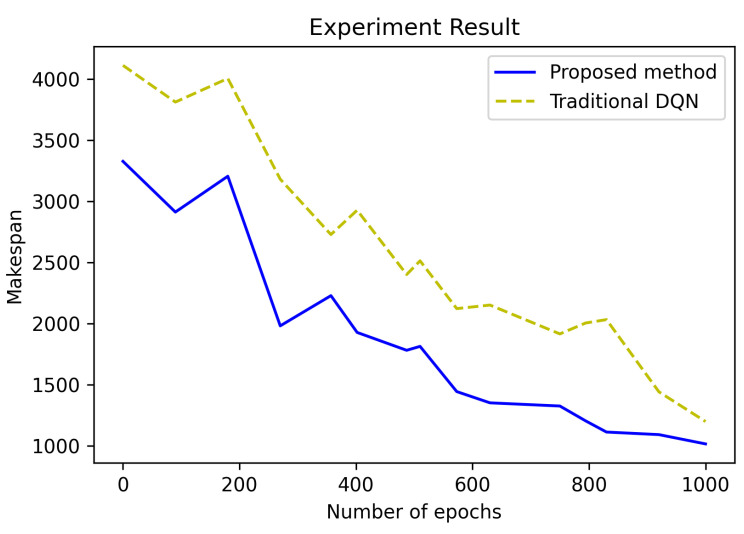
Comparison of the proposed method (DQN with transfer learning) with the existing DQN.

Figure 7 shows the result of comparing the convergence speed of the traditional DQN with the method of applying transfer learning to the proposed DQN. The values at M=10 and J=10 were compared. The proposed method is shown in Figure 7. It showed good performance. Solving JSPs is a major topic of research, but the way to solve JSPs is not just to improve performance through algorithms. The difference between the traditional DQN and the proposed DQN is that transfer learning was applied. In addition, there was a difference in processing divided by each area within the MEC structure.

**Figure 8:**
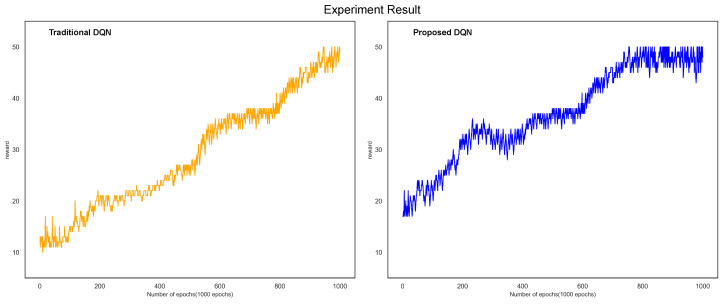
Comparison of the proposed method and the acquisition reward value of the traditional DQN.

In Figure 8, the value of the reward generated in the learning process of the traditional DQN and the proposed DQN showed an upward trend in both DQNs. The pattern in which the compensation converged indicated that the DQN model had an effect.

A reward occurs in performing reinforcement learning. When facing the problem, the student gives an answer, then the teacher checks student’s work and gives the reward for the student to fix the answer according to the reward. The purpose of reinforcement learning is to maximize future rewards. In the same way as the learning process of our experiment, we made it possible to improve performance by acquiring rewards.

In the case of the traditional DQN, the rewards generated in the learning process began to converge after 800 epochs and gradually appeared relatively stable after 900 iterations. In the case of the proposed DQN, it showed a shape in which the reward converged after 650 epochs and showed a more stable appearance than the traditional DQN after 700 iterations. In the case of the proposed DQN, it can be seen that the proposed DQN model was effective because it converged more rapidly than the traditional DQN.

**Figure 9:**
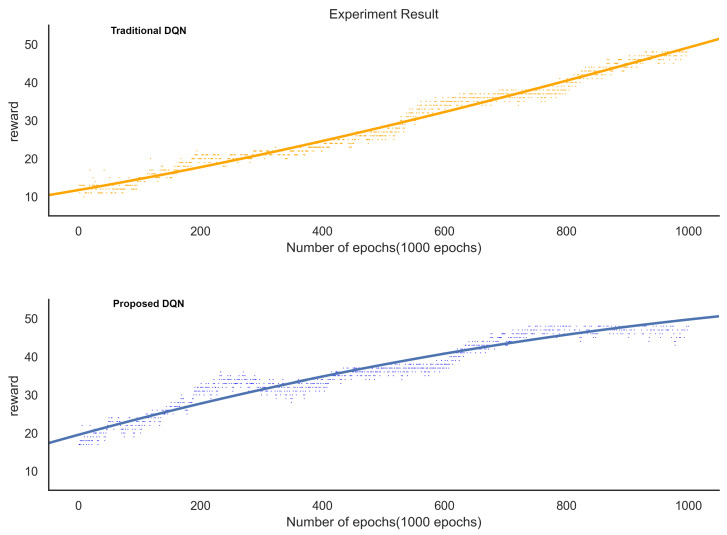
Comparison of the proposed method and the regression line of the conventional DQN.

Figure 9 shows the regression line for the graph of the compensation acquisition of the traditional and proposed DQNs. In the regression line, the estimated coefficients were 0.0530 and 0.0627, respectively. The dependent variable reward changed as the explanatory variable number of epochs increased by one. Accordingly, it can be seen that the traditional DQN increased by 0.0530 and the proposed DQN by 0.0627. Even by comparing the regression lines of the two graphs, it can be verified that the proposed DQN compensation acquisition proceeded faster. In addition, the *p*-value of the traditional DQN and proposed DQN was less than 0.001; hence, the regression model (regression equation) was significant.

Table 2 calculates the average value of the makespan matrix results of the DQN method, traditional DQN, SPT, and LPT of the proposed approach for dataset instances. The proposed DQN exhibited better performance for the target instance. This was a comparison of the experimental results for the proposed DQN method, the traditional DQN method, and the method using the dispatching rule.

### 5.3. Comparison of the Computing Methods

This section qualitatively compared the service based on the previously used computing methods with the service based on the proposed MEC-based scheduling framework. In general manufacturing companies, it is difficult for them to build and maintain their own manufacturing process systems in terms of cost, technology, and infrastructure. Therefore, we considered the viewpoints of customers receiving services from service providers, the viewpoints of solution distribution and operation of the service providers, and the scalability of the overall industry collaboration network. As an item for comparison, consider the service provider’s service scope, construction and maintenance cost, confidentiality, integrity, scalability, and support for collaboration with other service providers.

Table 3 compares the cloud, the existing cloud–edge hybrid, and the proposed MEC framework in terms of the services and frameworks. This indicated the utilization of the proposed method in the solution service. First, the service range was compared. The cloud was the narrowest because it was dependent on the company. In addition, in the existing hybrid method, the service range was wider than the cloud method, but eventually, the expansion of the computing service range becomes limited because it was dependent on the network cloud. The proposed method shared the core network in the process of cooperative scheduling with other companies, and therefore, the service range was the widest compared to the other two methods. If the service range were wide, there would be an added advantage that a wider variety of services could be provided to a larger number of users.

Although the overall cost of building a cloud framework is lower than that of edge computing, it is difficult for a small enterprise to bear the entire system construction and maintenance costs [48,49]. In addition, in the existing hybrid method, the cost for construction and maintenance is charged more than that of the cloud, but from the perspective of the customer, the cost is more active and the burden less. In the proposed method, scheduling was performed based on the provision of a solution previously produced by the service provider in the center. The service provider consumed a very high construction cost, but the MEC solution-based framework provided a low maintenance cost compared to the existing hybrid method. In addition, the cost consumption for service expansion through the core network was the lowest. From the customer’s point of view, the burden was less in terms of cost because it was incorporated into a framework that had already been established.

When comparing confidentiality, the cloud framework performed security tasks for all data centrally. Therefore, it had excellent security capability based on its strong computing power in the center. When a customer directly operates a cloud service, there is no risk regarding transferring corporate confidentiality to the service provider through its own network structure. However, if there is an issue with the security of the central cloud center, all infrastructure networks can be paralyzed. In the hybrid method, because the network is distributed to edge devices at the edge of the network, when the central cloud is attacked, the service can be maintained through the edge device. However, there is a direct security problem in edge devices owing to the weak computing power of the edge device [50]. Moreover, it is important to be aware of the sensitive problem that corporate secrets in the edge area are transferred to the cloud center [48]. The proposed method was located at the edge of a network composed of an MEC. The service provider handles only the overall solution flow, monitoring, and orchestration, excluding the customer’s sensitive information from the core network. Furthermore, even if the cloud center is attacked and the system is paralyzed, the damage to the service is minimized with a structure in which customers cooperate. Even if an attack enters the MEC, it can be resolved through collaborative offloading with the MEC itself and other neighboring MECs.

In terms of performance, cloud computing is the most powerful one. Because all computing resources are concentrated on the center, they can exhibit powerful performance. However, in terms of latency generation, communication latency with the machine may occur during the actual manufacturing process [51]. In the conventional hybrid method, the computing performance is lower than that performed in a single cloud, but it is compensated by the distribution of work between the cloud and edge devices. It has a significantly lower latency than cloud computing [52]. We could not reach a single cloud method to evaluate the performance of the proposed method. However, through a cooperative MEC framework that complements the relatively low computing performance of edge computing, collaborative devices in the network fill low computing resources. The latency is also very low at the edge of the network [53].

Finally, we examined whether it was possible to support collaboration with other customers. In a single cloud structure, service collaboration between partners is almost impossible. The central cloud server manages the system through a single-service network. Therefore, it is difficult to cooperate with other companies in terms of procedures, security, and distance. Compared to the cloud server, the hybrid computing method has the potential to collaborate with other customers, but similarly, there is a difficult part owing to the security problem of edge devices [54], especially the exposure of confidentiality between customers. In the scheduling domain of the smart factory manufacturing environment, research on working in collaboration with other service providers and other customers has not been conducted. The collaboration system we proposed was basically composed of a framework based on collaboration between MECs, so one can freely add other companies and new subscribers by opening the core network. In addition, service providers can easily form cooperative businesses through agreements between solution and service providers.

## 6. Conclusions

This paper proposed a smart manufacturing plant based on the MEC framework and conducted an experiment to solve the JSP from the perspective of the framework. Several edges (customers) cooperated under the supervision of a cloud center (service provider). This determined the work schedule for each complex system. In our study, we solved this problem by applying transfer learning to a DQN that combined DL and RL. As a result of the experiment, the proposed method showed a relatively better convergence effect than the conventional method in setting various parameters. Accordingly, it was proven that the method of applying efficient transfer learning to the DQN was superior to the existing DQN. When scheduling in the future manufacturing process, it would be much more complex and contain more of the company’s sensitive information. When the proposed DQN model is serviced from a service provider’s point of view, efficient scheduling would be feasible, and the company’s privacy would be guaranteed. Further, by connecting to the core network, customers can receive effective services not only in cooperation between customers in the service network, but also in the service range of other service providers.

The future challenge is to design different Q-table systems and compensation systems in RL and to improve the performance of the DQN model by adopting different NN types, as well as to improve the performance by applying a more advanced transfer learning method to the DQN. Comparing the performance of the reinforcement learning model designed by applying MEC and transfer learning in various ways with other previous studies is an important research task to be carried out in the future. In this study, the DQN was employed for the ease of implementation. Although advantageous for experimentation and model implementation, a better DRL model that has been studied recently can realize more detailed and higher performance scheduling results. Therefore, the future research scope is to find detailed scheduling results more effectively by developing the DQN method.

In addition, by applying network function virtualization (NFV), more MEC users can be attracted and safer cooperation between them can be guaranteed. This allows service providers to optimize maintenance costs and convenience. It is also interesting to design and implement an intelligent network management algorithm. The method can also be tested on a larger number of problem instances and experiments with performance by increasing the amount of learning. Finally, another future work is a quantitative comparison between our proposed MEC method and hybrid and cloud computing. This is an operation that compares various indicators such as communication time, latency, and computing speed. In particular, it is an important research topic to establish a reliable ecosystem between service providers and customers through intensive comparative analysis on security.

## Figures and Tables

**Table 1:** Description of the parameters.

Parameter	Description
Parameter for NN	64
Learning rate	0.001
Discount rate (γ)	0.7
Epsilon (ϵ)	0.9
Epsilon-difference (ϵ-diff)	0.02
Number of machines	According to the dataset setting
Number of jobs	According to the dataset setting

**Table 2:** Comparison table for the experimental results.

Instance	Proposed DQN	Traditional DQN	SPT	LPT
ft06	56	58	88	77
ft10	1016	1097	1074	1295
la13	1152	1239	1275	1230
la14	1215	1292	1427	1434
la15	1337	1339	1332	1612
orb1	1101	1211	1478	1410
orb2	993	1002	1175	1293
Average	981	1034	1121	1193

**Table 3:** Qualitative comparison in terms of the service provision of the existing and proposed structures.

Evaluation Index	Cloud	Cloud–Edge Hybrid	Proposed MEC
Service scope	Own company	One service provider	Many service providers
Service provider cost	Lowest	Highest	High
Customer cost	High	Low	Lowest
Security policy	Centralized security	Distributed security	Distributed security
Confidentiality	Not applicable	Confidential sharing risk	No confidential sharing
Performance	Strongest	Weakest	Filling the power
Latency	Highest	Low	Lowest
Cooperation	Not applicable	Difficulty	High usability

## Data Availability

Not applicable.

## References

[1] Zhang J., Ding G., Zou Y., Qin S., Fu J. (2019). Review of job shop scheduling research and its new perspectives under Industry 4.0. J. Intell. Manuf..

[2] Luo S. (2020). Dynamic scheduling for flexible job shop with new job insertions by deep reinforcement learning. Appl. Soft Comput..

[3] Yu W., Liang F., He X., Hatcher W.G., Lu C., Lin J., Yang X. (2017). A survey on the edge computing for the Internet of Things. IEEE Access.

[4] Angelopoulos A., Michailidis E.T., Nomikos N., Trakadas P., Hatziefremidis A., Voliotis S., Zahariadis T. (2020). Tackling faults in the industry 4.0 era—A survey of machine-learning solutions and key aspects. Sensors.

[5] Kuhnle A., Schäfer L., Stricker N., Lanza G. (2019). Design, implementation and evaluation of reinforcement learning for an adaptive order dispatching in job shop manufacturing systems. Procedia CIRP.

[6] Gu S., Lillicrap T., Sutskever I., Levine S. Continuous deep q-learning with model-based acceleration. Proceedings of the International Conference on Machine Learning.

[7] Mnih V., Kavukcuoglu K., Silver D., Rusu A.A., Veness J., Bellemare M.G., Graves A., Riedmiller M., Fidjeland A.K., Ostrovski G. (2015). Human-level control through deep reinforcement learning. Nature.

[8] Wei Y., Pan L., Liu S., Wu L., Meng X. (2018). DRL-scheduling: An intelligent QoS-aware job scheduling framework for applications in clouds. IEEE Access.

[9] Sharma P.K., Rathore S., Jeong Y.S., Park J.H. (2018). SoftEdgeNet: SDN based energy-efficient distributed network architecture for edge computing. IEEE Commun. Mag..

[10] Lin C.C., Deng D.J., Chih Y.L., Chiu H.T. (2019). Smart Manufacturing Scheduling With Edge Computing Using Multiclass Deep Q Network. IEEE Trans. Ind. Inform..

[11] Fortino G., Messina F., Rosaci D., Sarné G.M., Savaglio C. (2020). A trust-based team formation framework for mobile intelligence in smart factories. IEEE Trans. Ind. Inform..

[12] Moon J., Jeong J. Smart Manufacturing Scheduling System: DQN based on Cooperative Edge Computing. Proceedings of the 2021 15th International Conference on Ubiquitous Information Management and Communication (IMCOM).

[13] Liu J., Wan J., Zeng B., Wang Q., Song H., Qiu M. (2017). A scalable and quick-response software defined vehicular network assisted by mobile edge computing. IEEE Commun. Mag..

[14] Linthicum D. (2016). Responsive data architecture for the Internet of Things. Computer.

[15] Lin J., Yu W., Zhang N., Yang X., Zhang H., Zhao W. (2017). A survey on internet of things: Architecture, enabling technologies, security and privacy, and applications. IEEE Internet Things J..

[16] Dalla Cia M., Mason F., Peron D., Chiariotti F., Polese M., Mahmoodi T., Zorzi M., Zanella A. (2017). Using smart city data in 5G self-organizing networks. IEEE Internet Things J..

[17] Yan Y., Qian Y., Sharif H., Tipper D. (2012). A survey on cyber security for smart grid communications. IEEE Commun. Surv. Tutor..

[18] Satria D., Park D., Jo M. (2017). Recovery for overloaded mobile edge computing. Future Gener. Comput. Syst..

[19] Yan H., Li Y., Zhu X., Zhang D., Wang J., Chen H., Bao W. (2021). EASE: Energy-efficient task scheduling for edge computing under uncertain runtime and unstable communication conditions. Concurr. Comput. Pract. Exp..

[20] Shi W., Cao J., Zhang Q., Li Y., Xu L. (2016). Edge computing: Vision and challenges. IEEE Internet Things J..

[21] Frankston B. (2016). Mobile-Edge Computing versus The Internet?: Looking beyond the literal meaning of MEC. IEEE Consum. Electron. Mag..

[22] Mao Y., You C., Zhang J., Huang K., Letaief K.B. (2017). A survey on mobile edge computing: The communication perspective. IEEE Commun. Surv. Tutor..

[23] Vimal S., Khari M., Dey N., Crespo R.G., Robinson Y.H. (2020). Enhanced resource allocation in mobile edge computing using reinforcement learning based MOACO algorithm for IIOT. Comput. Commun..

[24] Demestichas P., Georgakopoulos A., Karvounas D., Tsagkaris K., Stavroulaki V., Lu J., Xiong C., Yao J. (2013). 5G on the horizon: Key challenges for the radio-access network. IEEE Veh. Technol. Mag..

[25] Khan W.Z., Ahmed E., Hakak S., Yaqoob I., Ahmed A. (2019). Edge computing: A survey. Future Gener. Comput. Syst..

[26] Charyyev B., Arslan E., Gunes M.H. Latency Comparison of Cloud Datacenters and Edge Servers. Proceedings of the IEEE Global Communications Conference (Globecom).

[27] Bonomi F., Milito R., Natarajan P., Zhu J. (2014). Fog computing: A platform for internet of things and analytics. Big Data and Internet of Things: A Roadmap for Smart Environments.

[28] Georgakopoulos D., Jayaraman P.P., Fazia M., Villari M., Ranjan R. (2016). Internet of Things and edge cloud computing roadmap for manufacturing. IEEE Cloud Comput..

[29] Evans D. (2011). The internet of things: How the next evolution of the internet is changing everything. CISCO White Pap..

[30] Yang X., Wang T., Ren X., Yu W. (2017). Survey on improving data utility in differentially private sequential data publishing. IEEE Trans. Big Data.

[31] Liu J., Mao Y., Zhang J., Letaief K.B. Delay-optimal computation task scheduling for mobile-edge computing systems. Proceedings of the 2016 IEEE International Symposium on Information Theory (ISIT).

[32] Sabella D., Vaillant A., Kuure P., Rauschenbach U., Giust F. (2016). Mobile-edge computing architecture: The role of MEC in the Internet of Things. IEEE Consum. Electron. Mag..

[33] Taleb T., Samdanis K., Mada B., Flinck H., Dutta S., Sabella D. (2017). On multi-access edge computing: A survey of the emerging 5G network edge cloud architecture and orchestration. IEEE Commun. Surv. Tutor..

[34] Morris I. (2016). ETSI Drops Mobile from MEC.

[35] Wang L., Hu X., Wang Y., Xu S., Ma S., Yang K., Liu Z., Wang W. (2021). Dynamic job-shop scheduling in smart manufacturing using deep reinforcement learning. Comput. Netw..

[36] Waschneck B., Reichstaller A., Belzner L., Altenmüller T., Bauernhansl T., Knapp A., Kyek A. Deep reinforcement learning for semiconductor production scheduling. Proceedings of the 2018 29th Annual SEMI Advanced Semiconductor Manufacturing Conference (ASMC).

[37] Moon J., Park G., Jeong J. (2021). POP-ON: Prediction of Process Using One-Way Language Model Based on NLP Approach. Appl. Sci..

[38] Wang Y. (2012). A new hybrid genetic algorithm for job shop scheduling problem. Comput. Oper. Res..

[39] Ge H.W., Sun L., Liang Y.C., Qian F. (2008). An effective PSO and AIS-based hybrid intelligent algorithm for job-shop scheduling. IEEE Trans. Syst. Man Cybern. Part A Syst. Hum..

[40] Tseng S.P., Tsai C.W., Chen J.L., Chiang M.C., Yang C.S. Job shop scheduling based on ACO with a hybrid solution construction strategy. Proceedings of the 2011 IEEE International Conference on Fuzzy Systems (FUZZ-IEEE 2011).

[41] Van Laarhoven P.J., Aarts E.H., Lenstra J.K. (1992). Job shop scheduling by simulated annealing. Oper. Res..

[42] Silver D., Huang A., Maddison C.J., Guez A., Sifre L., Van Den Driessche G., Schrittwieser J., Antonoglou I., Panneershelvam V., Lanctot M. (2016). Mastering the game of Go with deep neural networks and tree search. Nature.

[43] Mnih V., Kavukcuoglu K., Silver D., Graves A., Antonoglou I., Wierstra D., Riedmiller M. (2013). Playing atari with deep reinforcement learning. arXiv.

[44] Nguyen S., Zhang M., Tan K.C. (2016). Surrogate-assisted genetic programming with simplified models for automated design of dispatching rules. IEEE Trans. Cybern..

[45] Shiue Y.R., Lee K.C., Su C.T. (2018). Real-time scheduling for a smart factory using a reinforcement learning approach. Comput. Ind. Eng..

[46] Parisotto E., Ba J.L., Salakhutdinov R. (2015). Actor-mimic: Deep multitask and transfer reinforcement learning. arXiv.

[47] (2015). Jobshop Instance. http://jobshop.jjvh.nl/index.php.

[48] Sun W., Liu J., Yue Y. (2019). AI-enhanced offloading in edge computing: When machine learning meets industrial IoT. IEEE Netw..

[49] Ding S., Lin D. Dynamic Task Allocation for Cost-Efficient Edge Cloud Computing. Proceedings of the 2020 IEEE International Conference on Services Computing (SCC).

[50] Nath S.B., Gupta H., Chakraborty S., Ghosh S.K. (2018). A survey of fog computing and communication: Current researches and future directions. arXiv.

[51] O’donovan P., Gallagher C., Bruton K., O’Sullivan D.T. (2018). A fog computing industrial cyber-physical system for embedded low-latency machine learning Industry 4.0 applications. Manuf. Lett..

[52] Salaht F.A., Desprez F., Lebre A. (2020). An overview of service placement problem in Fog and Edge Computing. ACM Comput. Surv. (CSUR).

[53] Sodhro A.H., Pirbhulal S., De Albuquerque V.H.C. (2019). Artificial intelligence-driven mechanism for edge-computing-based industrial applications. IEEE Trans. Ind. Inform..

[54] Sarkar M., Banerjee S., Badr Y., Sangaiah A.K. (2017). Configuring a trusted cloud service model for smart city exploration using hybrid intelligence. Int. J. Ambient. Comput. Intell. (IJACI).

